# Dynamic Changes in Function and Proteomic Composition of Extracellular Vesicles from Hepatic Stellate Cells during Cellular Activation

**DOI:** 10.3390/cells9020290

**Published:** 2020-01-25

**Authors:** Xinlei Li, Ruju Chen, Sherri Kemper, David R Brigstock

**Affiliations:** 1 Center for Clinical and Translational Research, The Abigail Wexner Research Institute, Nationwide Children’s Hospital, Columbus, OH 43205, USA; Xinlei.Li@NationwideChildrens.org (X.L.); Ruju.Chen@NationwideChildrens.org (R.C.); Sherri.Kemper@Nationwidechildrens.org (S.K.); 2 Department of Surgery, Wexner Medical Center, The Ohio State University, Columbus, OH 43210, USA

**Keywords:** extracellular vesicle, hepatic stellate cell, hepatic fibrosis, exosome, microvesicle, liver

## Abstract

During chronic liver injury, hepatic stellate cells (HSC) undergo activation and are the principal cellular source of collagenous scar. In this study, we found that activation of mouse HSC (mHSC) was associated with a 4.5-fold increase in extracellular vesicle (EV) production and that fibrogenic gene expression (CCN2, Col1a1) was suppressed in Passage 1 (P1; activated) mHSC exposed to EVs from Day 4 (D4; relatively quiescent) mHSC but not to EVs from P1 mHSC. Conversely, gene expression (CCN2, Col1a1, αSMA) in D4 mHSC was stimulated by EVs from P1 mHSC but not by EVs from D4 mHSC. EVs from Day 4 mHSC contained only 46 proteins in which histones and keratins predominated, while EVs from P1 mHSC contained 337 proteins and these were principally associated with extracellular spaces or matrix, proteasome, collagens, vesicular transport, metabolic enzymes, ribosomes and chaperones. EVs from the activated LX-2 human HSC (hHSC) line also promoted fibrogenic gene expression in D4 mHSC in vitro and contained 524 proteins, many of which shared identity or had functional overlap with those in P1 mHSC EVs. The activation-associated changes in production, function and protein content of EVs from HSC likely contribute to the regulation of HSC function in vivo and to the fine-tuning of fibrogenic pathways in the liver.

## 1. Introduction

Hepatic fibrosis is the result of chronic liver injury and is characterized by the deposition of collagen and other insoluble extracellular matrix components [1]. The major fibrosis-causing cells in the liver are hepatic stellate cells (HSC) that reside in the Space of Disse [1,2,3]. Under normal conditions, HSC are quiescent pericyte-like cells that store large amounts of vitamin A in cytoplasmic lipid droplets. However, in response to liver injury. HSC undergo a phenotypic activation whereby they become contractile collagen-producing myofibroblasts that function either transiently in acute injury to produce a provisional matrix that supports hepatocyte repopulation or persistently in chronic injury to unrelentingly produce fibrotic material that causes scarring and exacerbates impaired liver function. These properties arise due to increased production and/or response by HSC of molecules involved in fibrogenesis, survival and inflammation [4]. The central role of HSC in fibrosis has spawned intense effort to target either key molecular mediators of fibrosis or the activated cells themselves (e.g., using cytotoxic agents) in a quest to develop new therapies for liver fibrosis [5,6]. While hepatic fibrosis is a major cause of morbidity and mortality and is a significant contributing factor to cirrhosis, which accounts for 32,000 deaths in the US and more than 1 million deaths globally each year [7], there is a severe lack of approved anti-fibrotics for improving patient outcomes [8,9]. Rational therapeutic strategies are being developed by leveraging our knowledge of the molecular mechanisms that regulate fibrosis and this has already led to the identification of a considerable number and variety of targets that are at various stages of pre-clinical or clinical testing [10,11]. Success in this area may most likely be achieved by combining anti-fibrotic strategies with other modalities that target hepatocyte injury or inflammation [10,12], but even so will require diligent design of clinical trials that have historically been vexed with difficulties and setbacks for reasons that include differences in fibrosis reversibility between humans and animal models, role of genetic background on fibrosis penetrance, protracted time-course of fibrosis development and influence of disease etiology on drug effectiveness [10,13,14]. Continued research of HSC biology is necessary to improve our understanding of the cellular and molecular aspects of fibrosis and to optimize development of new therapeutic options.

The biological activities of HSC occur as a result of their orchestrated interactions with other cells in the liver [12,15]. Recently, a new hepatic signaling network has been identified that involves the shuttling of molecular information between different liver cells by extracellular vesicles (EVs) [16,17,18,19,20]. EVs are membranous nanovesicles (50–500 nm) that comprise principally exosomes and microvesicles which are liberated from cells by, respectively, fusion of multivesicular bodies with the plasma membrane or budding off from the plasma membrane [21,22]. Despite many early reports that exosomes and microvesicles can be distinguished by size and/or the presence of marker proteins, it has recently been emphasized [23] that these features do not allow such discrimination. Thus, the use of the terms “exosome” or “microvesicle” firstly infers knowledge of specific EV biogenetic pathways that are extremely difficult to ascertain given current tools and technical limitations and, secondly, fails to take into account the innate heterogeneity of EVs in biological samples. Accordingly, since current recommendations for operationally defining EVs include reference to the cell of origin [23], in this report we have used “EVs from HSC” or ‘HSC EVs” to describe the population of EVs that we recovered from HSC-conditioned culture medium using the stated enrichment methods. Irrespective of the precise EV subtypes involved, accumulating data suggest that a complex EV network exists in the liver whereby quiescent or activated HSC exchange EV cargo molecules (proteins, mRNAs, microRNAs) with one another or with cells such as hepatocytes or liver sinusoidal endothelial cells [24,25,26,27,28,29,30,31,32]. This bi-directional molecular transfer ensures that normal homeostatic functions or injury-associated responses in the liver are fine-tuned and synchronized at the cellular level.

An area of growing interest is the manner in which the EV cargo content varies according to the phenotypic status of their cells of origin. In view of the highly distinct functions of quiescent versus activated HSC, we hypothesized that the EVs from these cells exhibit fundamental differences in their biological properties and/or molecular payload. In this report we provide evidence for such differences in terms of their respective actions on cultured HSC and their protein cargo as assessed by mass spectrometry and proteomic analysis. We also provide a comparative proteomic analysis of EVs from activated mouse and human HSC and show substantial overlap in their constituent protein content and their predicted functional roles.

## 2. Material and Methods

### 2.1. Animals and Primary Mouse HSC (mHSC) Culture

Animal procedures were conducted using protocol 04504AR approved by the Institutional Animal Care and use Committee of the Research Institute at Nationwide Children’s Hospital (Columbus, OH, USA). Mouse HSC (mHSC) isolation was accomplished using standard procedures. Briefly, wild-type male Swiss Webster mice (4–5 wks old; *n* = 5) underwent systemic perfusion with 20–30 mL HBSS under deep anesthesia followed by two steps of perfusion with 15 mL Dulbecco’s Modified Eagle’s Medium (DMEM) containing 2 mg/mL pronase or 0.5 mg/mL collagenase I, respectively. After this 40 min perfusion technique, livers were removed, diced with scissors and digested in 0.33 mg/mL pronase and 166 U/mL DNase I in DMEM at 37 °C for 10 min. mHSC were collected by Optiprep density centrifugation, counted using a hemocytometer, and plated at 10^6^ cells/mL in DMEM/F12 medium containing 20% fetal bovine serum for 1 h. For short-term culture, primary mHSC were switched to serum-free Hyclone^TM^ SFM4MegaVir medium (Thermo Fisher Scientific, Waltham, MA, USA) one day after initial seeding and the medium was collected 3 days later (i.e., on Day 4) for EV isolation. For long-term culture, the medium on Day 1 primary mHSC was replaced with fresh DMEM/F12/fetal bovine serum (FBS) and the cells were cultured until Day 10 whereupon they were split 1:5 and the resulting passage 1 (P1) cells were grown in DMEM/F12/FBS cells for 5 days. On Day 15, the medium was removed and replaced with SFM4MegaVir medium which was then collected 2 days later (i.e., on Day 17) for EV isolation.

### 2.2. Mouse HSC Characterization

D4 or P1 mHSC cultures were analyzed for auto-fluorescence upon excitation at 405 nm and emission at 450 nm to detect vitamin A-positive oil droplets using a LSM 510 confocal microscope (Carl Zeiss Microscopy Inc., Thornwood, NY, USA) and the incidence of positive cells was established by comparison to the number of cells observed with the same instrument by bright field microscopy. mHSC were fixed with 4% paraformaldehyde for 15 min at room temperature and incubated with anti-αSMA (1:500; Invitrogen, Waltham MA) or anti-reelin (1:200; R&D Systems, Minneapolis, MN, USA) for 1 h and developed with, respectively, Alexa Fluor 488 goat anti-mouse IgG (Invitrogen) or Alexa Fluor 568 donkey anti-goat IgG (Invitrogen). The frequency of αSMA- or reelin positive cells was determined by comparison to the number of cells that were positive for DAPI nuclear staining Quantitative real time polymerase chain reaction (qRT-PCR) was used to evaluate gene expression in primary mHSC within 4 days of plating or after passage to P1 as described below.

### 2.3. Human LX-2 CELLS

The human HSC (hHSC) line, LX-2, (a kind gift from Dr Scott Friedman, Icahn School of Medicine at Mount Sinai, New York, NY, USA) was cultured in DMEM/10% FBS. Cells were seeded at 10^7^ cells/mL and allowed to grow to 90% confluency. For harvesting of EVs, spent medium was removed and replaced with serum-free DMEM which was then collected 2 days later.

### 2.4. EV Isolation

EVs from primary mHSC or human LX-2 hHSC were enriched from their respective serum-free conditioned media by sequential centrifugation of the supernatant obtained after 300× *g* for 15 min, 2000× *g* for 20 min, 10,000× *g* for 30 min and ultracentrifugation at 100,000× *g* for 70 min at 4 °C (T-70 i fixed-angle rotor; Beckman Coulter, Brea, CA, US, USA). The pellet from the latter step was dispersed in phosphate-buffered saline (PBS) and underwent a repeat round of ultracentrifugation. The use of differential ultracentrifugation is consistent with current recommendations for EV enrichment and is the technique most commonly used by researchers for primary EV separation and concentration [23] The resulting pellet was dispersed in PBS and the constituent EVs were characterized as described below.

### 2.5. Nanoparticle Tracking Analysis (NTA)

A Nanosight 300 instrument (Malvern Instruments, Westborough, MA) was calibrated with 100 nm polystyrene latex microspheres (Magsphere Inc., Pasadena, CA, USA) and used to analyze HSC EVs that were diluted to 10^7^–10^8^ particles/mL in PBS. Each EV sample was analyzed in duplicate and mean particle concentration and size were estimated with v3.2 analytical software (Malvern Instruments). Recordings were performed at room temperature with a camera gain of 15 and a shutter speed of 4.13 ms. The detection threshold was set to 6.

### 2.6. Western Blotting

Proteins were detergent-extracted from EVs and subjected to sodium dodecyl polyacrylamide gel electrophoresis (SDS-PAGE; 10 µg/lane) and transferred to nitrocellulose membrane for Western blot detection using antibodies to CD9 (1:500; Abcam, Cambridge, MA, USA), flotillin-1 (1:200; BD Biosciences, San Jose, CA, USA), Tsg101 (1:500; Thermo Fisher Scientific), fibronectin (FN1) (1:1000; Abcam), FN1 EDA domain (1:250; Sirius Biotech, Genoa, Italy), FN1 EDB domain (1:100; Sirius Biotech), pan-keratin (1:500; Cell Signaling Technology, Danvers, MA, USA), proteasome subunit alpha type-6 (PSMA6, 1:500; ABclonal Inc, Woburn, MA, USA) or ribosomal protein S27a (RPS27A, 1:500; ABclonal). Blots were developed using IRDye 800 CW secondary antibodies (LI-COR Biosciences, Lincoln, NE, USA) in conjunction with Odyssey CLx Imaging (LI-COR Biosciences).

### 2.7. Gene Expression in HSC

Gene expression was evaluated in primary mHSC on Day 4 or after passage to P1. To assess EV bioactivity, D4 or P1 mHSC were switched overnight to medium containing 2% exosome-depleted serum and then treated for 48 h with 8 µg/mL EV from D4 mHSC, P4 mHSC, or LX-2 hHSC. Total RNA was extracted from the cells using a miRNeasy mini kit (Qiagen) and reverse transcribed using a miScript II RT kit (Qiagen) according to the manufacturer’s protocols. Transcripts for common fibrosis-related molecules (e.g., collagen 1a1, cellular communication network factor 2 (CCN2), αSMA) or that corresponded to proteins which were determined by MS to be differentially expressed in EVs from D4 versus P1 mHSC (see below) were evaluated by qRT-PCR using an Eppendorf Mastercycler System and SYBR Green Master Mix (Eppendorf, Hauppauge, NY). Each reaction was run in duplicate, and samples were normalized to 18S ribosomal RNA. Negative controls were a non-reverse transcriptase reaction and a non-sample reaction. Primers are shown in Supplemental Table S1.

### 2.8. EV Protein Extraction and Digestion

EV pellets were resuspended in 100 µL 50 mM ammonium bicarbonate containing 0.1% Rapigest (Waters Corp., Milford, MA, USA), sonicated twice for 10 s and incubated with shaking for 1 h at room temperature. Extracts were clarified at 13,000 rpm in a microcentrifuge and protein concentration was determined using a Qubit assay kit (Thermo Fisher Scientific). Dithiothreitol was added to a final concentration of 5 mM and the sample was incubated at 65 °C for 30 min. Iodoacetamide was then added to a final concentration of 15 mM and the sample was incubated in the dark, at room temperature for 30 min. Sequencing grade trypsin (Promega Corp, Madison, WI, USA) was added at a 1:30 ratio and the sample was digested overnight at 37 °C. Trifluoroacetic acid was then added to a final concentration of 0.5% and sample was incubated at 37 °C for 30 min to precipitate the Rapigest. The sample was clarified at 13,000 rpm for 5 min in a microcentrifuge, dried in a vacufuge and resuspended in 20 µL 50 mM acetic acid. Peptide concentration was determined at 280 nm using a nanodrop spectrophotometer (Thermo Fisher). Five separate D4 EV preparations and three separate P1 EV preparations, all from different mHSC isolations, were individually prepared in this manner for mass spectrometry.

### 2.9. Mass Spectrometry (MS)

EV protein identification was performed using nano-liquid chromatography-nanospray tandem mass spectrometry (LC/MS/MS) on a Thermo Scientific Q Exactive mass spectrometer equipped with an EASY-Spray™ Sources operated in positive ion mode. Each sample was injected into a µ-Precolumn Cartridge (Thermo Scientific) and desalted with 0.1% formic acid in water for 5 min. Samples were then separated on an easy spray nano column (Pepmap^TM^ RSLC, C18 3µ 100 A, 75 µm × 150 mm, Thermo Scientific) using a two-dimensional (2D) RSLC high-performance liquid chromatography system (Thermo Scientific). Mobile phase A was 0.1% formic acid in water and mobile phase B was acetonitrile/0.1% formic acid. Peptide elution was achieved with a multi-linear gradient comprising 2–35% B over 225 min, 35–55% B over 35 min, 55–90% B over 10 min and then 90% B isocratic for 5 min. After each run, the column was equilibrated in 2% B for 20 min before the next sample injection.

The MS/MS method was a Top10 method: the analysis was programmed for a full scan recorded between *m*/*z* 400–1600 and an MS/MS scan to generate product ion spectra to determine amino acid sequence in consecutive scans starting from the most abundant peaks in the spectrum, and then selecting the next nine most abundant peaks. To achieve high mass accuracy MS determination, the full scan was performed at a resolution setting of 70,000. The AGC target ion number for full scan was set at 3 × 10^6^ ions, maximum ion injection time was set at 100 ms. MS/MS was performed using a stepped normalized CE of 25, 30, and 35, acquired at a resolution of 17,500 with an AGC target of 1 × 10^5^ ions and a maximum injection time of 50 ms. Dynamic exclusion was enabled with a repeat count of 1 within 30 s.

Sequence information from the MS/MS data was processed by converting the raw files into a merged file (.mgf) using MS convert (ProteoWizard). The resulting mgf files were searched using Mascot Daemon by Matrix Science version 2.6.0 (Boston, MA, USA) and the database searched against Uniprot Mouse database. The mass accuracy of the precursor ions was set to 10 ppm, and accidental inclusion of 1 13C peaks was also included into the search. The fragment mass tolerance was set to 0.05 Da. Four missed cleavages for the enzyme were permitted. A decoy database was also searched to determine the false discovery rate (FDR) and peptides were filtered according to the FDR. Proteins with less than 1% FDR as well as a minimal of two significant peptides detected were considered as valid proteins. Proteomics data was summarized in scaffold to allow for spectral counting analysis. Complete MS datasets are available in the Supplemental Data section as Tables S2–S4.

### 2.10. Gene Ontology, Pathway Enrichment, and Protein-Protein Interaction Networks

Grouping of EV proteins into cellular components was accomplished using Gene Ontology (GO) analysis (http://geneontology.org/) and the Funrich database (https://FunRich.org). EV protein functions and utilities were determined using DAVID ((https://david.ncifcrf.gov/) analysis of The Kyoto Encyclopedia of Genes and Genomes (KEGG; https://www.genome.jp/kegg/). Search Tool for the Retrieval of Interacting Genes (STRING, https://string-db.org/) was employed to determine interactions among EV proteins using a minimum interaction score of 0.4 for the D4 mHSC EV dataset, and 0.9 for the P1 mHSC or LX-2 EV datasets. The Markov Cluster Algorithm method with an inflation parameter of 2 was applied for clustering. These analyses were each performed with a criterion FDR < 0.05.

### 2.11. Statistical Analysis

Experiments were performed at least twice in duplicate, with data expressed as mean ± S.E.M. qRT-PCR data were analyzed by student’s *t*-test using Sigma plot 11.0 software (SPSS Inc., Chicago, IL, USA). A *p* value < 0.05 was considered statistically significant.

## 3. Results

### 3.1. Characterization of Primary mHSC

**Figure 1:**
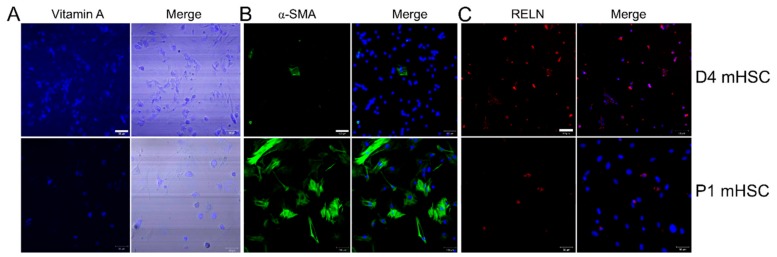
Characterization of primary mouse HSC. (**A**) Primary D4 or P1 mHSC were evaluated for vitamin A autofluorescence (left) or examined by phase contrast (right), and imaged by confocal microscopy. Based on six fields, Vitamin A was positive in 91.3 ± 3.4% of D4 mHSC and 85.4 ± 8.0% of P1 mHSC. (**B**) D4 or P1 mHSC were stained with anti-αSMA (left) which was then merged with DAPI (right). Based on three fields, α SMA was positive in 3.3 ± 0.7% of D4 mHSC and 93.3 ± 5.0% of P1 mHSC. (**C**) D4 or P1 mHSC were stained with anti-reelin (left) which was then merged with DAP1 (right). Based on two fields, the frequency of reelin-positive cells was 98.4 ± 1.6% for D4 mHSC and 13.1 ± 1.2% for P1 mHSC. Bars: 50 µm for A and C; 100 µm for B.

Autofluorescence for vitamin A, which is stored in cytoplasmic oil droplets in HSC and is useful for identifying or characterizing HSC, was detected in 91.3 ± 3.4% of D4 mouse cells and 85.4 ± 8.0% of P1 mouse cells (Figure 1A). Staining for αSMA, a marker of HSC activation, was detectable in only 3.3 ± 0.7% of D4 cells but in 93.3 ± 5.0% of P1 cells demonstrating the high degree of activation of P1 cells as compared to D4 cells (Figure 1B). Staining for reelin, which is preferentially expressed in quiescent HSC but not in other hepatic cell types [33], was detected in 98.4 ± 1.6% of D4 mHSC and 13.1 ± 1.2% of P1 mHSC (Figure 1C). This is consistent with previous findings showing that reelin expression declines during HSC activation [33] and also argues that our early (and hence later) primary HSC cultures were essentially devoid of contaminating cells that might otherwise confound analysis. Since absolute quiescence is not feasible because HSC start to autonomously activate after isolation, the above staining data nonetheless led us to conclude that D4 (the earliest time point at which we could reproducibly harvest sufficient numbers of EVs for detailed analysis) represented a relatively quiescent state as compared to P1 cells which were highly activated, thus validating our subsequent comparative EV analysis.

### 3.2. Physico-Chemical and Biological Properties of EVs from mHSC or hHSC

**Figure 2:**
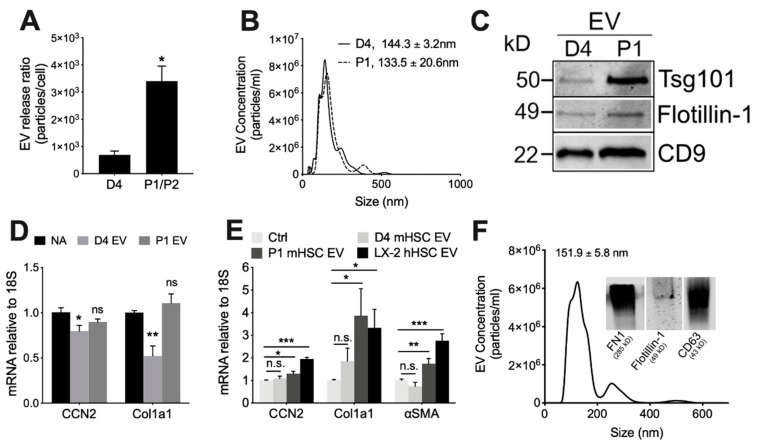
Characterization of EVs from mouse HSC. NTA was performed on EVs that had been purified by differential centrifugation of 2-day serum-free conditioned medium from D4 HSC or P1-P2 HSC, with particle number expressed as a function of (**A**) cell number or (**B**) particle size (mean ± S.E.M. for particle diameter (nm) is indicated). (**C**) Western blot analysis of D4 or P1 EVs (25 µg EV protein per lane) showing the presence of common EV proteins. (**D**) P1 HSC or (**E**) D4 HSC were treated for 48 h with 8 µg/mL EVs from D4 mHSC, P1 mHSC or LX-2 hHSC after which expression for the indicated transcripts was determined by qRT-PCR. (**F**) EVs purified from LX-2 hHSC under serum-free conditions were analyzed by NTA or Western blot (inset). *, *p* < 0.05; **, *p* < 0.01; ***, *p* < 0.005; n.s., not significant.

Serial low speed centrifugation and ultracentrifugation of serum-free medium from each cell type resulted in a significantly greater yield (4.5-fold) of EVs from P1-P2 mHSC than D4 mHSC showing that EV production was positively correlated with mHSC activation (Figure 2A). EVs from D4 mHSC or P1 mHSC were very similar in terms of mean size (144.3 ± 3.2 nm and 133.5 ± 20.61 nm, respectively) and size range (approx. 50–500 nm) (Figure 2B). Western blot revealed that EVs from either D4 or P1 mHSC were positive for proteins such as CD9, Tsg101 and flotillin-1 that are commonly associated with or enriched in EVs from other systems (Figure 2C). Thus, EVs from D4 or P1 mHSC exhibited shared physical properties and expression of key EV proteins. Additional characterization of mHSC EVs including conventional or cryogenic transmission electron microscopy and dynamic light scattering have been reported by us previously [25].

We have previously demonstrated and characterized the binding interactions between target HSC and EVs from mHSC or hHSC, including functional delivery of specific EV payload components [24,25,27,28,34]. When added to P1 mHSC, EVs from D4 mHSC caused expression of CCN2 or Col1a1 to be suppressed in the cells (Figure 2D), an observation that is consistent with our earlier reports that EVs from quiescent mHSC suppress fibrogenic gene expression as well as levels of proteins such as αSMA [25,27,28]. We extended these findings by showing that EVs from P1 mHSC did not alter expression of these genes in P1 mHSC (Figure 2D), yet they stimulated expression of CCN2, Col1a1 or αSMA in D4 mHSC (Figure 2E), the latter of which is consistent with our prior demonstration that αSMA mRNA or protein in HSC is stimulated by EVs from activated HSC or by EVs carrying an enriched CCN2 payload [24]. By contrast, EVs from D4 mHSC had no significant effect on gene expression in D4 mHSC (Figure 2E). We previously conducted transmission electron microscopy and cell binding assays of EVs purified from LX-2 hHSC [24,25] and expanded those data in this study by using NTA to show that they were of similar size as those from mHSC (151.9 ± 5.8 nm; size range 50–500 nm), contained EV-associated proteins such as flotillin-1 and CD63 (Figure 2F), and, like EVs from P1 mHSC, were able to stimulate gene expression in D4 mHSC (Figure 2E).

### 3.3. Proteomic Analysis of EVs from mHSC

**Figure 3:**
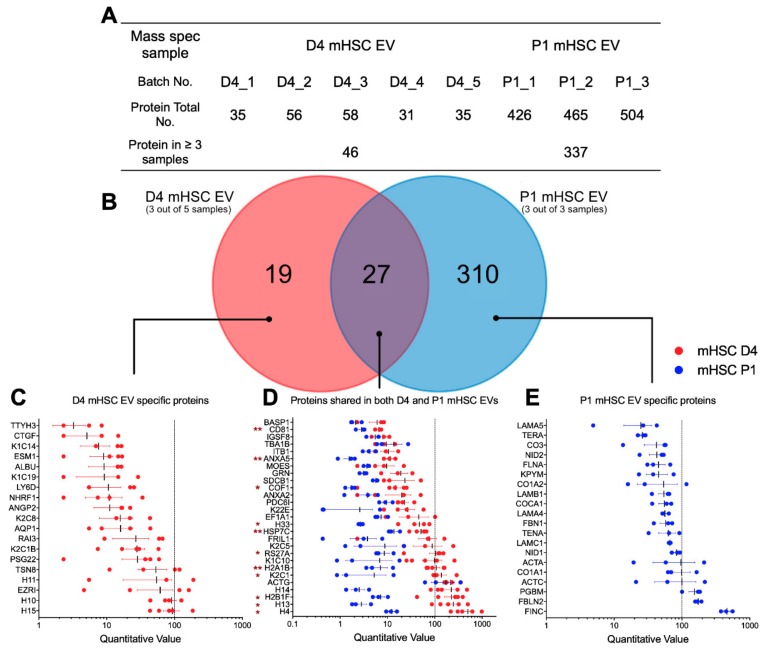
Proteomic composition of mHSC EVs. (**A**) Summary of quantitative features of EV proteins analyzed from five D4 mHSC EV samples or three P1 mHSC EV samples. (**B**) Venn diagram showing distribution of proteins between EVs from D4 versus P1 mHSC. The figure also shows the identities and quantifications of (**C**) the 19 proteins specific for D4 mHSC EVs; (**D**) the 27 proteins shared by both groups for which 11 proteins were significantly enriched in EVs from D4 mHSC (*, *p* < 0.05, **, *p* < 0.01) and (**E**) the 20 most abundant proteins specific for EVs from P1 mHSC.

**Figure 4:**
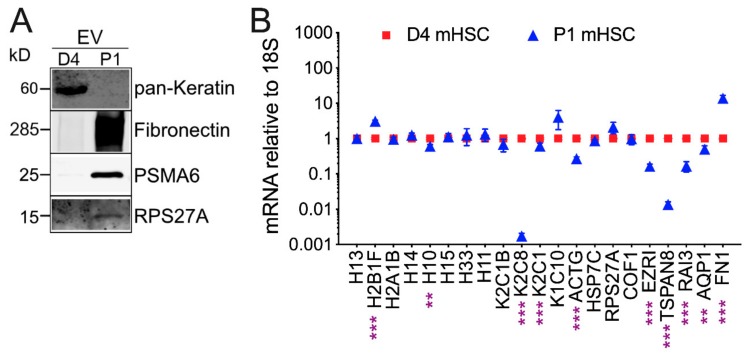
EV Western blot and cellular transcript levels for differentially expressed EV proteins. (**A**) Western blots of EVs from D4 mHSC or P1 mHSC to verify differential levels of representative proteins identified by MS. (**B**) qRT-PCR was performed on RNA from D4 or P1 mHSC using primers designed to amplify mRNA corresponding to proteins that were more highly expressed in EVs from D4 mHSC than in EVs from P1 mHSC (see Figure 3C,D). Data are mean ± S.E.M. for duplicate determinations performed twice individually. **, *p* < 0.01, ***, *p* < 0.005.

Mass spectrometry for mHSC EVs revealed striking qualitative and quantitative differences of proteins in EVs from D4 mHSC versus EVs from P1 mHSC (Supplemental Tables S2 and S3). Analysis of five separate EV samples from D4 mHSC or three separate EV samples from P1 mHSC resulted in the identification of between, respectively, 31 and 58 proteins or 426 and 504 proteins (Figure 3A). Subsequent analysis of these data was focused on 46 proteins that were present in at least three EV samples from D4 mHSC and on 337 proteins that were common to all 3 EV samples from P1 mHSC. Of these, 19 proteins were unique to D4 mHSC EVs, 310 proteins were unique to P1 mHSC EVs and 27 proteins were shared between D4 and P1 mHSC EVs (Figure 3B). With respect to the 19 D4-specific proteins, the most abundant (quantitative value ~100) included three histones (H15, H10, H11) and ezrin (EZR1), a membrane-bound cytoskeleton linker protein (Figure 3C). Of the shared proteins, 11 proteins were present at significantly higher levels in EVs from D4 mHSC compared to EVs from P1 mHSC and six of these (H4, H13, H2B1F, H14, K2C1, H2A1B) were the most abundant proteins (quantitative value ~100–1000) in EVs from D4 mHSC (Figure 3D). No D4 mHSC-specific EV protein had this level of abundance (Figure 3C) but three proteins (FN1 (also termed FINC), FBLN2, PGBM) reached a comparable abundance level among the proteins specific to P1 mHSC EVs (Figure 3E). Western blot analysis was used to confirm the high abundance of keratin in EVs from D4 mHSC and of FN1 in EVs from P1 mHSC (Figure 4A). Western blot and MS analysis demonstrated the presence of EDA and EDB sequences in the FN1 protein showing that it was the cell-associated form as opposed to the plasma form which lacks these domains (Supplemental Figure S1). For the 21 proteins that were differentially expressed in EVs from D4 versus P1 cells, analysis of the producer cells by RT-PCR showed that their corresponding cellular transcripts were comparably differentially expressed for only eight candidates (H10, K2C8, K2C1, ACTG, EZR1, TSPAN8, RAI3, AQP1) with the rest showing no significant difference in expression except H2B1F which showed inverse cellular mRNA expression (P1 > D4) as compared to EV protein level (D4 > P1) (Figure 4B).

**Figure 5:**
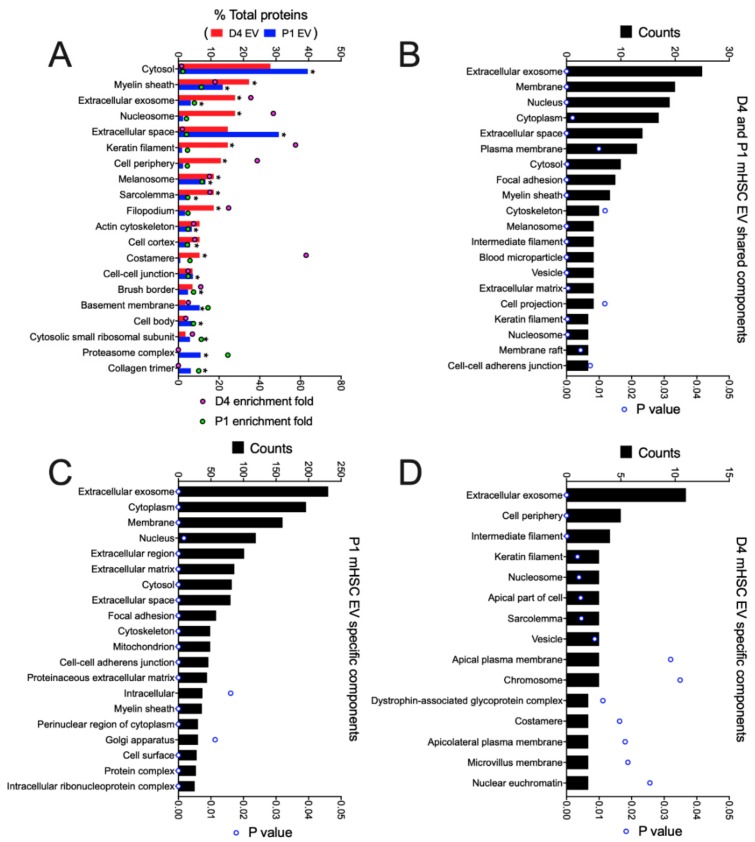
Cellular component analysis of proteins in EVs from D4 and/or P1 mHSC. GO analysis was performed using the Funrich database to identify EV proteins with significant enrichment versus the Uniprot database. The figure shows the 20 most highly ranked cellular components for proteins that were (**A**) in the entire mHSC EV proteome from D4 cells compared to those from P1 cells; (**B**) shared between EVs from D4 and P1 mHSC; (**C**) specific to P1 mHSC EVs. (**D**) Components specific to D4 mHSC EVs. Only cellular components with significant enrichment (* *p* < 0.05) are shown.

When all D4 mHSC EV proteins or all P1 mHSC EV proteins were analyzed using GO/Funrich, the 20 most highly represented components were generally differentially expressed, with proteasome complex and collagen trimer being unique to P1 mHSC EVs (Figure 5A). The 27 proteins common to EVs from both D4 and P1 mHSC shared enrichment for components that included exosomes, membranes, cytoplasm, extracellular space, focal adhesion, ECM, microparticles, vesicles, cytoskeleton and cell-cell adherins (Figure 5B), many of which were shared with proteins that were P1-specific (Figure 5C). Proteins in D4-specific EVs included some of the same components (exosomes, vesicles) but in there was an absence of extracellular or adhesion components and instead a concentration of membraneous (apical, aipicolateral or microvillus membrane, sarcolemma), nuclear (chromosome, euchromatin) and structural-functional (dystrophin glycoprotein complex, costamere) components (Figure 5D).

**Figure 6:**
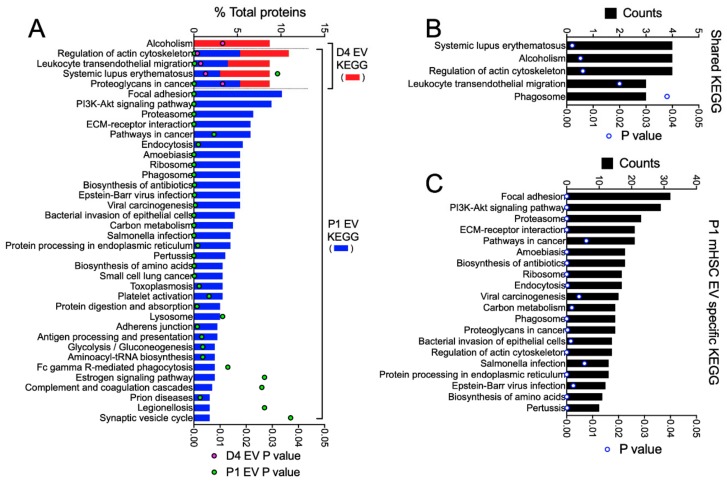
KEGG pathway analysis of proteins in EVs from D4 and/or P1 mHSC. EV proteins were analyzed online using DAVID v6.8 software for KEGG pathway analysis. The figure shows pathways that were (**A**) in the entire mHSC EV proteome from D4 mHSC versus P1 mHSC; (**B**) shared between EVs from D4 and P1 mHSC; or (**C**) specific to P1 mHSC EVs (top 20 pathways shown). Only pathways with significant enrichment (*p* < 0.05) are shown.

KEGG pathway analysis of all 46 proteins in D4 mHSC EVs and all 337 proteins in P1 mHSC EVs revealed one unique pathway for D4 mHSC EVs (alcoholism, involving 8% of the proteins), four shared pathways for D4 and P1 mHSC EVs (regulation of actin cytoskeleton, leucocyte transendothelial migration, systemic lupus erythematosus, and proteoglycans in cancer, each involving 3–11% of the proteins) and 33 unique pathways for P1 mHSC EVs (for which focal adhesion, PI3K-Akt signaling, proteasome, ECM-receptor interaction and pathways in cancer each involved the highest proportion (8–10%) of the proteins) (Figure 6A). While no KEGG pathway was identified for the 19 D4 mHSC-specific EV proteins, most of the these pathways were, respectively, prominent for either the 27 proteins that were shared between D4 and P1 mHSC EVs (regulation of actin cytoskeleton, leucocyte transendothelial migration, systemic lupus erythematosus, alcoholism) (Figure 6B) or the P1 mHSC EV-specific proteins (focal adhesion, PI3K-Akt signaling, proteasome, ECM-interactions, pathways in cancer) (Figure 6C).

STRING analysis of the proteomic data to identify principal protein interactions and functions revealed striking differences between each type of EV. Whereas the proteins in D4 mHSC EVs were organized into a simple network comprising nodes that included keratins and histones (Supplemental Figure S2), those in P1 mHSC EVs demonstrated much more complex interactions with principal nodes containing proteins associated with collagens, ECM, vesicular transport, metabolic enzymes, proteasomes, ribosomes, chaperones and tRNA ligase (Supplemental Figure S3). Representative proteins from key nodes in each network were verified by Western blot as being specific for D4 mHSC EVs (keratin) or P1 mHSC EVs (PSMA6, RPS27A) (Figure 4A).

### 3.4. Proteomic Analysis of EVs from hHSC

**Figure 7:**
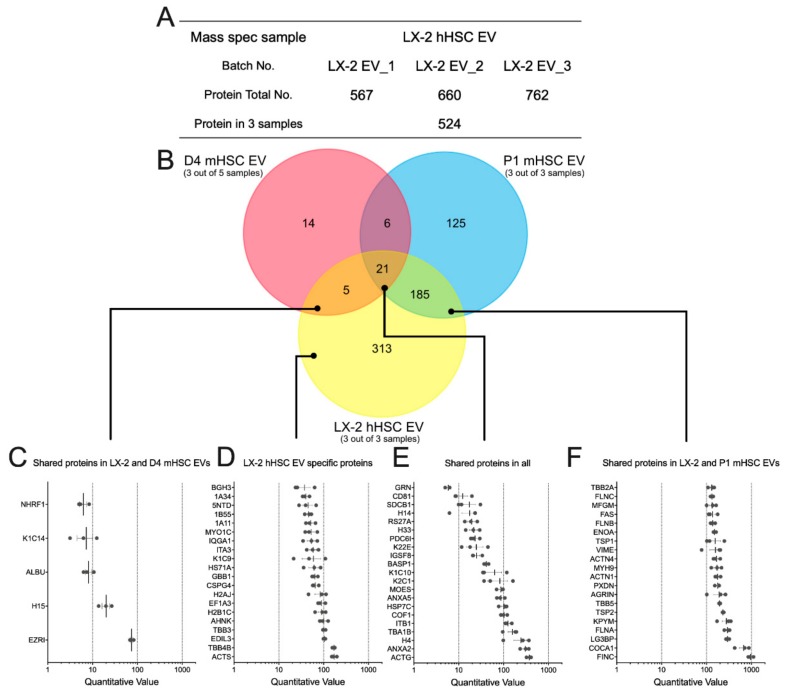
Proteomic composition of EVs from LX-2 hHSC and/or D4 or P1 mHSC. (**A**) Summary of quantitative features of EV proteins analyzed from LX-2 hHSC EV samples. (**B**) Venn diagram showing distribution of proteins between EVs from LX-2 hHSC, D4 mHSC and P1 mHSC. Also shown are the identities and quantifications of (**C**) the five proteins shared between LX-2 hHSC EVs and D4 mHSC EVs; (**D**) the 20 most abundant proteins specific to EVs from LX-2 hHSC; (**E**) the 21 proteins shared by EVs from all three cell types; and (**F**) the 20 most abundant proteins shared by EVs from LX-2 hHSC and P1 mHSC.

We next used MS to investigate the protein components in EVs from LX-2 hHSC (Supplemental Table S4). Analysis of three separate LX-2 hHSC EV samples resulted in the identification of between 567 and 762 proteins, 524 of which were common to all three samples (Figure 7A). In light of the profibrogenic actions of EVs from activated mHSC or hHSC (Figure 2E), it was interesting that of the 524 LX-2 hHSC EV proteins, 206 were shared with the 337 proteins in EVs from P1 mHSC (Figure 7B). Only 26 LX-2 hHSC proteins were shared with EVs from D4 mHSC, (Figure 7B). All three types of EVs had 21 proteins in common with one another (Figure 7B). Quantitative analysis (Figure 7C–F) showed that when proteins in each group were ranked based on expression levels in LX-2 EVs, those shared with EVs from D4 mHSC but not P1 mHSC had the lowest expression (five proteins; quantitative value range = 8–80), while those shared with EVs from P1 mHSC but not with EVs from D4 mHSC had the highest expression (185 proteins; quantitative range = 100–1000 for the top 20) (Figure 7C,F). The most abundant protein was FN1 (Figure 7F), the presence of which was confirmed in LX-2 hHSC EVs by Western blot analysis (Figure 2F). Top-ranked proteins specific to LX-2 hHSC EVs had an intermediate level of expression (313 proteins; quantitative value 50–200 for the top 20) while the proteins in LX-2 hHSC EVs that were shared with EVs from both D4 and P1 mHSC were very variably expressed (21 proteins; quantitative range 5–500) (Figure 7D,E).

**Figure 8:**
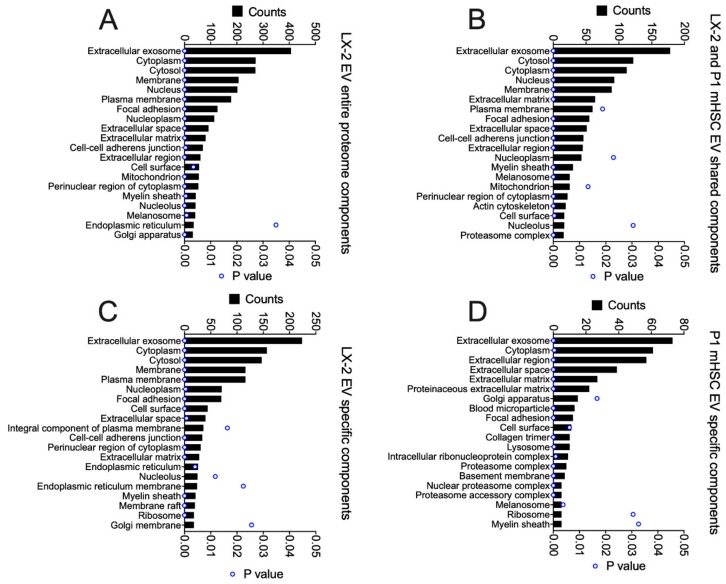
Cellular component analysis of proteins in EVs from LX-2 hHSC and/or P1 mHSC. GO analysis was performed on proteins in EVs from LX-2 hHSC as in Figure 5. The figure shows the 20 most highly ranked cellular components for proteins that were (**A**) in the entire LX-2 HSC EV proteome; (**B**) shared between EVs from LX-2 hHSC and P1 mHSC; (**C**) specific to LX-2 hHSC EVs; or (**D**) specific to P1 mHSC EVs. Only cellular components with significant enrichment (*p* < 0.05) are shown.

**Figure 9:**
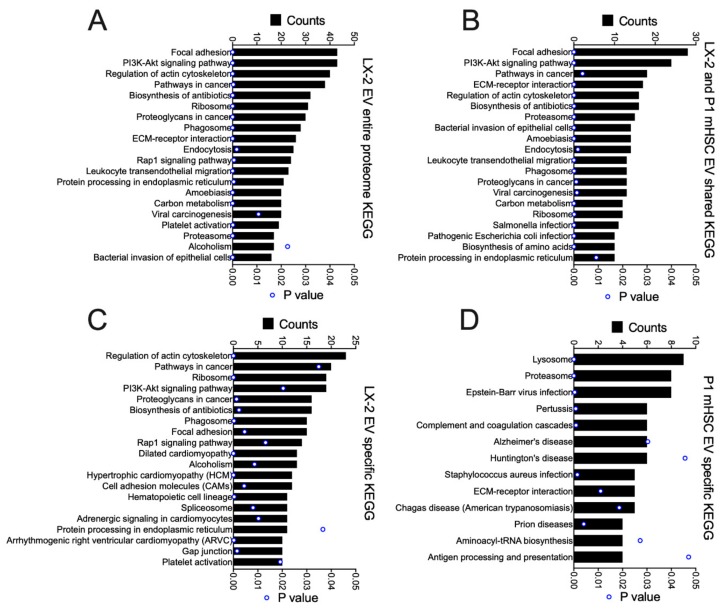
KEGG pathway analysis of proteins in EVs from LX-2 hHSC and/or P1 mHSC. EV proteins were analyzed as in Figure 6. The figure shows the top 20 pathways identified for (**A**) the entire LX-2 hHSC EV proteome; (**B**) proteins shared between LX-2 hHSC EVs and P1 mHSC EVs; or (**C**) proteins unique to LX-2 hHSC EVs. (**D**) Pathways identified for EV proteins that were specific to P1 mHSC. Only pathways with significant enrichment (*p* < 0.05) are shown.

GO analysis was performed on the total proteins identified in EVs from activated human or mouse HSC, as well as those that were shared or not shared between them (Figure 8). For the entire EV proteome from LX-2 cells, the principal components were related to exosome, cytoplasm, cytosol, membrane, nucleus, ECM, focal adhesion, extracellular space, cell surface, cell-cell adherins, perinuclear region of cytoplasm, mitochondrion, nucleoplasm and myelin sheath (Figure 8A). Many of these components were shared with P1 mHSC EVs but additional components in this shared group included melansome, actin cytoskeleton and proteosome complex (Figure 8B). The same or highly similar components were represented by the proteins that were unique to EVs from either LX-2 cells (Figure 8C) or P1 mHSC (Figure 8D). The outcome of this component analysis was largely reflected in the KEGG analysis of the respective EV protein groups (Figure 9). For example, the top 20 KEGG pathways for the complete LX-2 hHSC EV proteome (Figure 9A) showed substantial overlap with the pathways for the P1 mHSC EV proteome (Figure 6A) and included focal adhesion, PI3K-Akt signaling, regulation of actin cytoskeleton, pathways in cancer, proteoglycans in cancer, ribosome, phagosome, leukocyte transendothelial migration, protein processing in the ER and carbon metabolism. These pathways were also predominant for the 206 proteins shared between LX-2 hHSC EVs and P1 mHSC EVs (Figure 9B). Some of the same or related components were evident for LX-2 hHSC EV-specific proteins (regulation of cytoskeleton, pathways in cancer, ribosome, PI3k-AKT signaling, proteoglycans in cancer, phagosome, focal adhesion, cell adhesion molecules, alcoholism) (Figure 9C) or for P1 mHSC EV-specific proteins (proteasome, ECM-receptor interactions) (Figure 9D) but the remaining components in these groups were quite dissimilar and diverged from those typically noted above. Finally, STRING analysis of the entire LX-2 hHSC EV proteome (Supplemental Figure S4) or of the EV proteins shared between LX-2 cells and P1 mHSC (Supplemental Figure S5) revealed major interactions between nodes associated with collagens, ECM, metabolic enzymes, vesicular transport, chaperones or ribosomes and these were very similar to those for the entire P1 mHSC EV proteome (Supplemental Figure S3).

## 4. Discussion

EVs have attracted considerable attention for the role they likely play in mediating cell-cell communication throughout the body. In the liver, EVs are proposed to regulate hepatic homeostasis or to contribute to pathophysiogical processes such as viral spread, non-alcoholic steatohepatitis, alcoholic liver disease, and cancer [16,17]. A large body of research has shown that hepatocytes infected with hepatitis B or C viruses or that have been exposed to agents such as alcohol, carbon tetrachloride or palmitate produce EVs that stimulate macrophage activation and immune function which are common features of numerous liver diseases and often associated with fibrotic pathology [30,35,36,37,38,39,40]. HSC fibrogenesis is stimulated by these types of hepatocyte-derived EVs [29,32,41], while other phenotypic features of activated HSC such as migration and AKT phosphorylation have been shown to be enhanced by EVs from liver sinusoidal endothelial cells [42]. On the other hand, EVs from healthy hepatocytes [43], various stem cells [44,45,46,47,48,49,50,51] or the serum of healthy mice [52] have the ability to inhibit experimental liver fibrosis, largely by suppressing inflammatory responses and/or pathways of activation or fibrogenesis in HSC. The recognition that HSC are EV targets has highlighted an important new mechanism by which fibrogenic pathways in the liver are modulated and has given a new lead for novel anti-fibrotic therapies based on suppressing the action of pro-fibrotic EVs or harnessing the actions of EVs that are intrinsically anti-fibrotic.

As shown in this report, HSC themselves are also EV producers but relatively little is known about this phenomenon. Whereas the physico-chemical properties of HSC EVs were quite consistent whether their producer cells were activated or not, we found that the HSC EV production rate, biological properties and protein composition were highly dependent on HSC activation status. Specifically, our results showed, firstly, that fibrogenic gene expression was suppressed upon exposure of activated HSC to EVs from relatively quiescent HSC but not to EVs from activated HSC and, secondly, that gene expression in relatively quiescent HSC was stimulated upon exposure of the cells to EVs from activated HSC but not to EVs from relatively quiescent cells. Thus, pathways of EV communication between HSC may be either stimulatory or inhibitory and are manifest principally when the activation status of the EV producer HSC is different to that of the EV recipient HSC. We also showed that HSC activation is associated with a 4.5-fold increase in EV release and that EVs from activated HSC contained considerably more proteomic information than their quiescent counterparts: there were 337 proteins in EVs from P1 mHSC but only 46 proteins in EVs from D4 mHSC. Generally, EVs from D4 mHSC exhibited a high abundance and high proportion of histones and keratins, while EVs from P1 mHSC contained proteins principally associated with extracellular spaces, ECM, proteasome complexes, collagens, ECM, vesicular transport, metabolic enzymes, ribosomes and chaperones. These differences reflected the distinct phenotypes and functions of their respective EV producer cells: quiescent HSC are resting vitamin-A storing cells whereas activated HSC are contractile myofibroblasts that interact with various immune cells, are highly proliferative and migratory, are metabolically very active, have high energy requirements and produce numerous cytokines, chemokines and extracellular matrix components. Even so, it will be important in future studies to evaluate the molecular payload of EVs from HSC that have been activated in vitro by cytokines (e.g., TGF-β) or in vivo due to liver injury as there may be qualitative or quantitative differences that have functional impact as compared to the EVs in this study that were from HSC that had autonomously activated in culture.

It is interesting that FN1 was expressed exclusively in EVs from P1 HSC and was the most abundant protein overall in EVs from activated or quiescent HSC. FN1 exists either as a soluble plasma form that lacks EDA and EDB domains and is produced principally by hepatocytes or as a cell-associated form which contains the EDA and EDB domains and is produced by numerous cell types. Analysis of the FN1 protein in P1 mHSC EVs showed it to be the cell-associated form because sequencing identified a near-complete EDA domain as well as a partial N-terminal EDB domain, consistent with its detection using FN1 antibodies directed to the EDA or EDB regions. We have previously reported that HSC EVs use cell surface integrins as receptors, including integrin α5β1, which is a receptor for FN1 [34]. In activated HSC, FN1-integrin α5β1 interactions are important for the regulation of cell adhesion, survival, cytoskeletal rearrangements or expression of matrix metalloproteases or collagen I [53,54,55,56,57], but this interaction may also underlie the binding of EVs to target HSC, the extent of which will be dependent on activation-associated changes in cellular integrin expression and EV FN1 levels. Importantly, EV FN1 may also directly participate in downstream pro-fibrogenic actions of HSC EVs in light of recent studies showing, firstly, that FN1 in cancer cell microvesicles mediates their ability to confer transformation characteristics on fibroblasts and epithelial cells [58] and, secondly, that exosomal FN1 mediates the mitogenic activity of exosomes from mesenchymal stem cells [59]. Such functions may also be conserved in EVs from hHSC, since we showed FN1 to be the most abundant component of EVs from LX-2 cells in these studies. Indeed, an important feature that emerged from the current investigation was the recognition that EVs from activated hHSC have the same pro-fibrogenic properties and share many of the same proteins as their mouse counterparts resulting in considerable overlap in the components and pathways in which they are involved. The 206 proteins that were common to both species represented approximately 40% of all 524 proteins in EVs from activated hHSC and 61% of all 337 proteins in EVs from activated mHSC. The incorporation of these shared proteins into EVs from activated HSC of mice and humans suggests that their functional roles were under strong selective pressure during evolution.

It remains to be determined what other constituents (protein, RNA or miRNA or combinations thereof) in the EV molecular payload might be relevant to the relative suppressive or stimulatory actions of EVs from, respectively, quiescent or activated HSC. Even so, it is striking that the proteomic payloads of EVs from activated human or mouse HSC correspond to cellular components that are well characterized for their involvement in HSC activation and/or fibrogenesis (i.e., ECM, proteasome complexes, collagens, metabolic enzymes, ribosomes, chaperones) and it is tempting to speculate that the EV counterparts have similar functions. Although we have previously used a transfection approach to show that HSC-derived EVs can shuttle GFP-CCN2 intercellularly [24], this overexpression system may not have faithfully mimicked native mechanisms because CCN2 was not detected in EVs from P1 mHSC in this study. With respect to the anti-fibrogenic actions of EVs from D4 mHSC, the high prevalence of keratins or histones in the relatively small proteome suggests that structural elements or nucleosomal regulation may underlie the suppressive activities. However, preliminary transfection studies in which activated HSC were transfected with cDNAs encoding single D4 mHSC-specific or -enriched EV histones (H4, H10, H11, H13, H14, H15, H2B1F) did not result in an attenuation of fibrogenic markers even though each over-expressed protein was detected by Western blot (X.L. and D.R.B., unpublished data), suggesting that these proteins are not anti-fibrogenic at least when tested individually, but additional combinatorial testing of these and other candidates (e.g., keratins) must be undertaken in the future. For a variety of differentially expressed mHSC EV proteins, we found that the levels of their corresponding cellular transcripts were quite variable, ranging from concordant to discordant with their respective EV protein levels, suggesting that there is selectivity in the post-transcriptional or post-translational mechanisms by which a given protein is incorporated into the EVs.

Apart from proteins, the EV molecular payload contains miRs and mRNAs that may also contribute to EV-mediated regulation of fibrogenesis. For example, we previously showed that inhibition of pro-fibrogenic CCN2 in quiescent HSC is achieved by targeting of the CCN2 3′ untranslated region by Twist-1-miR-199a-miR-214 and that all three components of this axis can be delivered in EVs to activated HSC in which CCN2 expression is then suppressed [25,27,28]. Even so, one must recognize that while these types of reductionist strategies to understand EV bioactivity have been widely pursued in the EV research field in general, they do not take account of EV heterogeneity at the single vesicle and systems level and the importance of adopting a more global view of EV cargo as it relates to functional aspects of EV biology has recently been emphasized [60]. Thus, a holistic approach may be preferable for understanding the combinatorial actions of EV cargo constituents in mediating EV biological actions [60]. We expect that detailed comparative studies between EVs from quiescent versus activated HSC of their respective miRnomes and RNAnomes, together with their proteomes as accomplished in this study, will provide a foundation for the identification of EV components and their corresponding cellular targets that accounts for their distinct biological actions. Whatever the factors involved, it is interesting to speculate that the suppressive actions of EVs from quiescent HSC may help to protect the liver from overt HSC activation, especially in cases of mild or acute injury.

In conclusion, mHSC activation is associated with an increase in EV production, a switch in EV bioactivity whereby EV-mediated suppression of mHSC fibrogenic gene expression gives way to EV-mediated stimulation of the same, and a dramatic increase in the complexity of the EV proteome. Activated hHSC produce EVs that exhibit profibrogenic activities and have similar protein components and functions as EVs from activated mHSC. We thus propose that activation-associated changes in production, function and protein content of EVs from HSC may contribute to the regulation of HSC function in vivo and to the fine-tuning of fibrogenic pathways in the liver.

## Supplementary Materials

The following are available online at https://www.mdpi.com/2073-4409/9/2/290/s1: Figure S1. Fibronectin in EVs from P1 mHSC; Figure S2. String analysis for entire proteome in EVs from D4 mHSC; Figure S3. String analysis for entire proteome in EVs from P1 mHSC; Figure S4. String analysis for entire proteome in EVs from LX-2 hHSC; Figure S5. String analysis for proteins shared in EVs from P1 mHSC and LX-2 hHSC; Table S1. qRT-PCR primers used for mouse gene detection; Table S2. MS datasets for D4 mHSC proteome; Table S3. MS datasets for P1 mHSC proteome; Table S4. MS datasets for LX-2 hHSC proteome.
