# Supplementary figures and images for: Dynamic Changes in Function and Proteomic Composition of Extracellular Vesicles from Hepatic Stellate Cells during Cellular Activation
Source: Cells. 2020 Jan 25;9(2):290. doi: 10.3390/cells9020290 (PMC7072607; doi:10.3390/cells9020290)

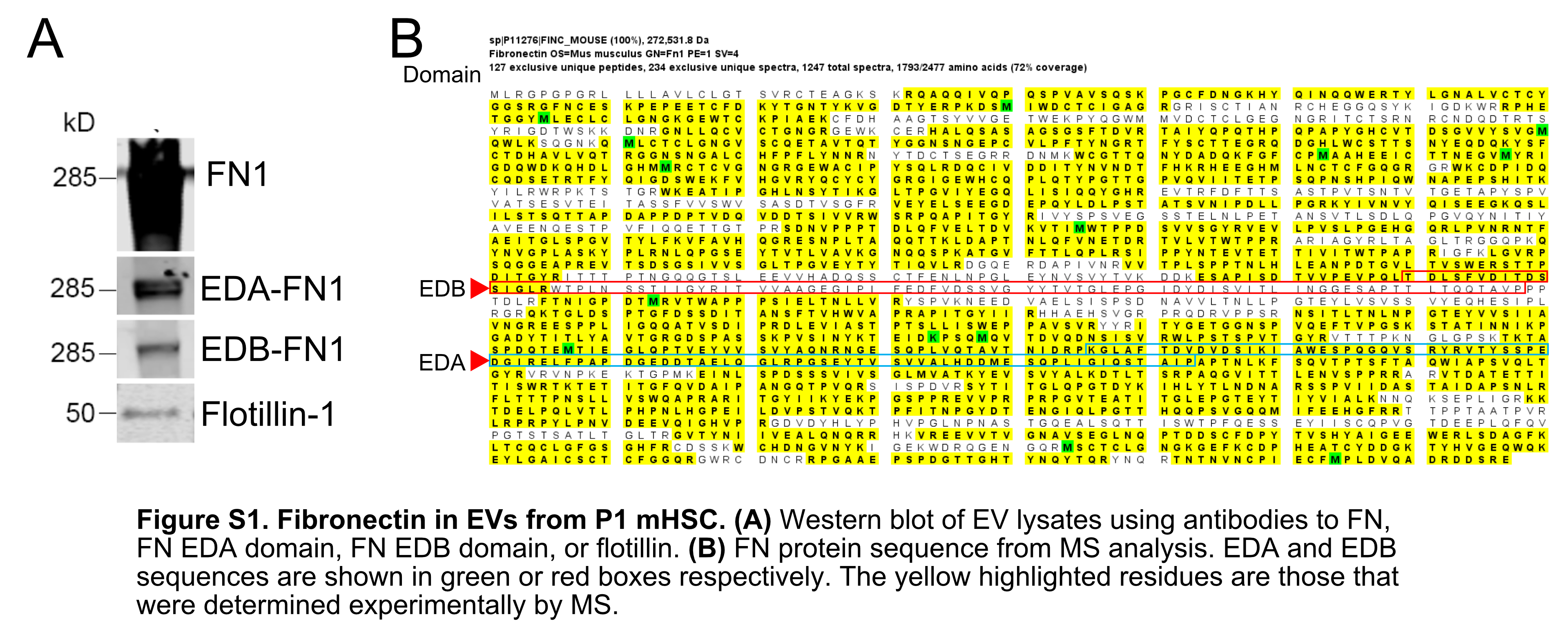

Supplement: Supplementary file 1 [file cells-09-00290-s001.zip › Suppl Fig 1(010820).tiff]

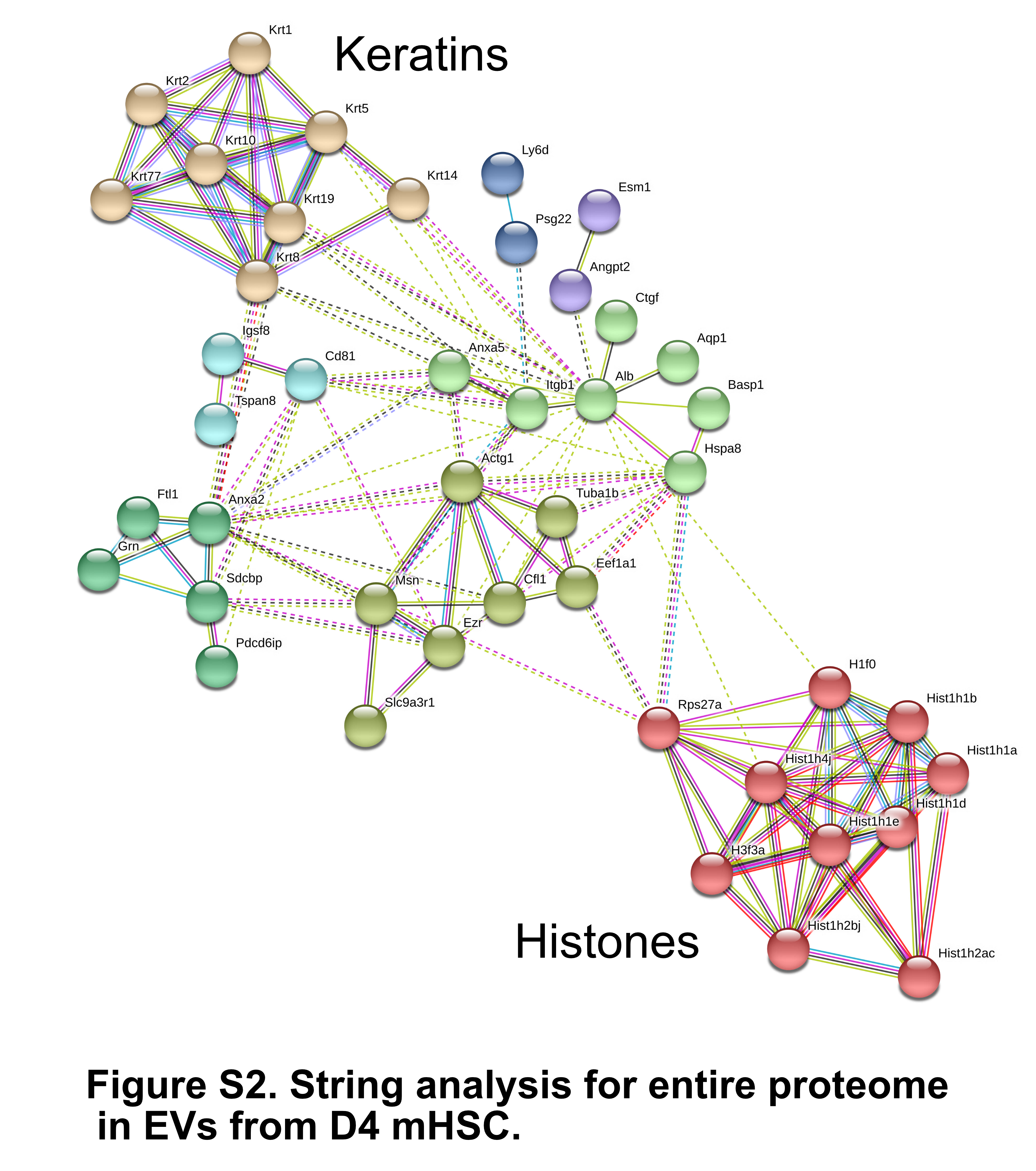

Supplement: Supplementary file 1 [file cells-09-00290-s001.zip › Suppl Fig 2(010820).tiff]

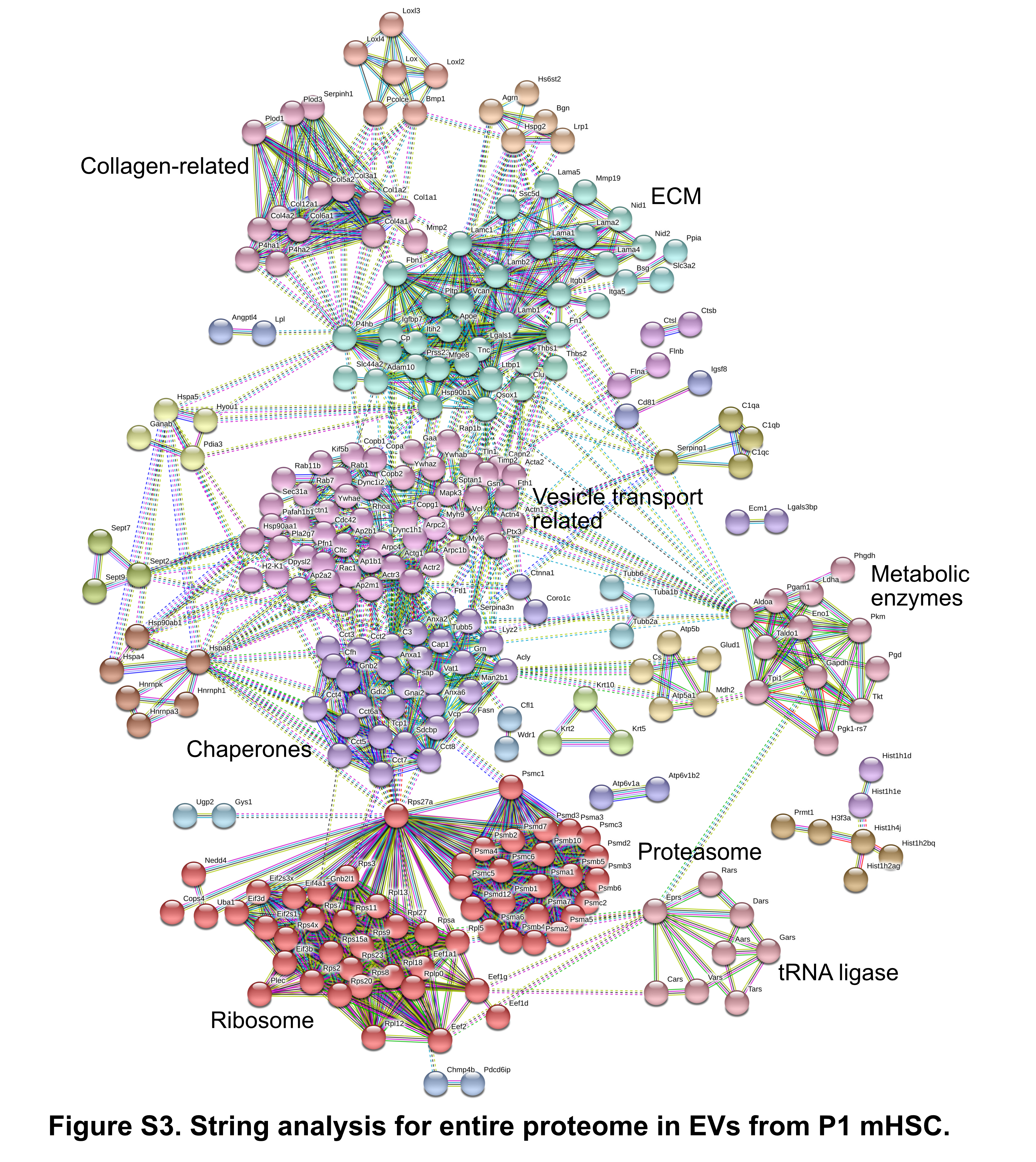

Supplement: Supplementary file 1 [file cells-09-00290-s001.zip › Suppl Fig3(010820).tiff]

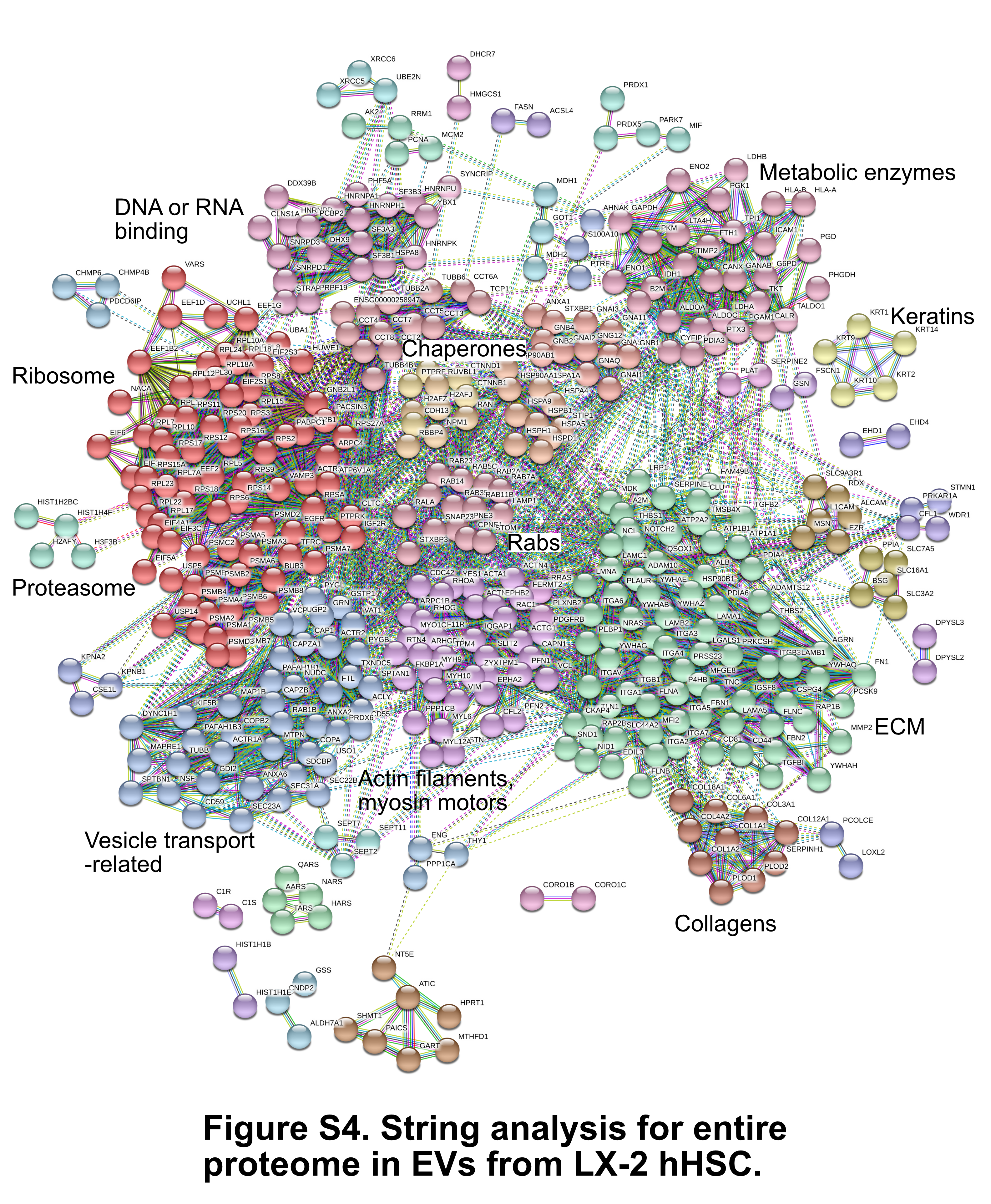

Supplement: Supplementary file 1 [file cells-09-00290-s001.zip › Suppl Fig4(010820).tiff]

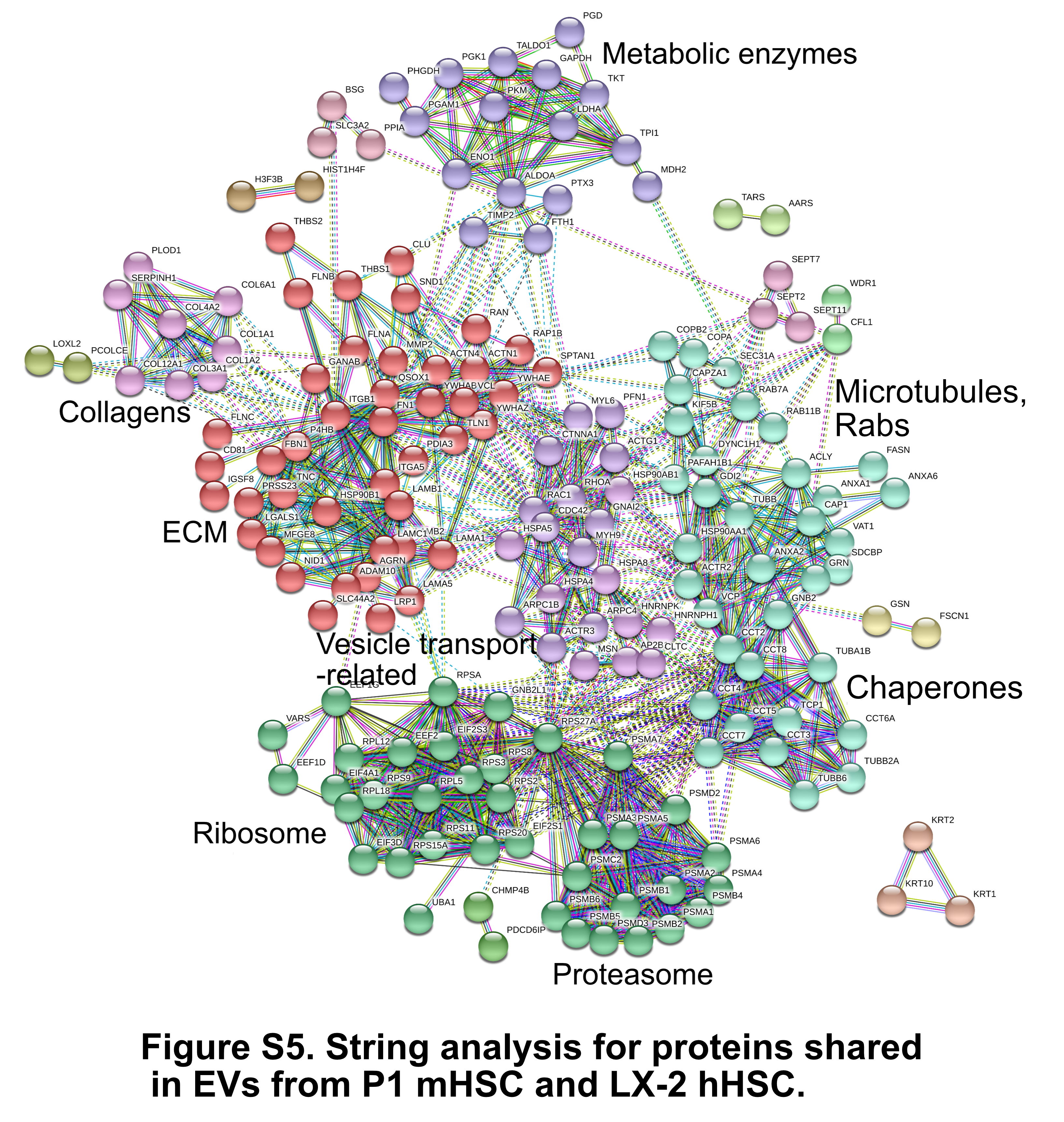

Supplement: Supplementary file 1 [file cells-09-00290-s001.zip › Suppl Fig5(010820).tiff]
